# Prevalence of Certain Urogenital Bacterial *Mollicutes* in Patients Suffering from Infertility

**DOI:** 10.1155/2022/2812788

**Published:** 2022-03-22

**Authors:** Motasem Y. Al-Masri, Intesar Khaleel Ashour, Ashraf Swafta, Sami Al-Shunar

**Affiliations:** ^1^Division of Pathology and Medical Laboratory Science, Department of Biomedical Sciences, An-Najah National University, Nablus, West Bank, State of Palestine; ^2^Department of Biology and Biotechnology, An-Najah National University, Nablus, West Bank, State of Palestine; ^3^Al-Shunar Infertility Center, Nablus, West Bank, State of Palestine

## Abstract

**Introduction:**

*Mollicutes* urogenital tract infections are considered a possible cause of infertility worldwide. Genital *Mollicutes* infections are difficult and impractical to diagnose by culturing or serology. *Mollicutes* included in this study were *Mycoplasma hominis*, *Ureaplasma urealyticum*, and *Mycoplasma genitalium*. This cross-sectional study aimed to determine the prevalence of *M. hominis*, *U. urealyticum*, and *M. genitalium* genital infections among infertile males and females patients.

**Methods:**

This study included 103 patients who visited Al-Shunar Clinic in Nablus city in Palestine and diagnosed with infertility during January 2018 to October 2018. The semen, urine, and/or vaginal swab specimens collected from patients were examined by PCR for detection of *M. hominis*, *U. urealyticum*, and *M. genitalium*.

**Results:**

A total of 57 semen, 37 urine, and 16 vaginal swab specimens were collected. Out of the 110 examined specimens, 35 (31.8%) were PCR positive for at least one *Mollicutes*, which were 16 (14.6%) *M. hominis*, 11 (10%) *U. urealyticum*, and 8 (7.3%) *M. genitalium*. Significant association were found between infections of *M. hominis* and *U. urealyticum* (*P*=0.044) and between *M. hominis* and *M. genitalium* (*P*=0.005) infections. *M. hominis* infection was found in significantly (*P*=0.048) higher percentage in males (20.6%) in comparison with females (5.7%). On the other hand, *M. genitalium* infection rate in females (8.6%) was slightly higher than males (7.4%). *M. hominis* was more prevalent in all age groups except for patient's age group 40–50 years old, where *M. genitalium* was more prevalent. *M. hominis* was also more prevalent in all occupation types and among all smokers.

**Conclusion:**

Urogenital infections caused by *M. hominis*, *M. genitalium*, and *U. urealyticum* could be a possible cause of infertility among patients with different age groups, genders, and occupations. Thus, more attention by infertility centers and physicians is required in adopting molecular methods for diagnosis of infections by these microorganisms.

## 1. Introduction

Infertility is a disease of the reproductive system that is defined by failure to achieve a clinical pregnancy after 12 months of regular unprotected sexual intercourse [1]. About 70 million couples worldwide were reported to suffer from infertility [2].

According to World Health Organization (WHO) guidelines, the absence of a prior history of pregnancy indicates the presence of primary infertility. On the other hand, individuals are diagnosed with secondary infertility when they have a prior pregnancy but are suffering from infertility latter [2, 3].

Infertility of men and women results from several factors. These factors include congenital disorders, hormonal disorders, physical problems (e.g., varicocele and changes caused by chronic and acute genital tract infections), lifestyle problems, environmental hazards, and psychological states. All of these factors can lead to impairments in the function of genital organs, reproductive cells production, semen quality, sperm cell transport to the oocyte, fertilization, and embryo implantation steps [4–6].

Of the most importantly recorded etiologic agents of infertility were infections of reproductive tract, in particular sexually transmitted diseases (STDs) and *tuberculosis* complications [7]. Among *Mollicutes,* both *Ureaplasma* and *Mycoplasma* species are transmitted through sexual intercourse and are associated with male urethritis and prostatitis, as well as female urethritis, vaginosis, and cervix inflammation [8], which might affect human fertility [5, 6].

*Mycoplasmas* are discussed as ones of the important etiological factors of male infertility [9]. In addition to that, their male urogenital tract infections are often asymptotic. Asymptotic presence of these microorganisms in urogenital tracts may have a negative influence on male reproductive health [10].

Pelvic inflammatory disease (PID) has a multifactorial etiology, and it is strongly reflected on the health of women's reproductive tract. There is evidence for the association of *Mycoplasma genitalium* as well as other *Mycoplasma* and *Ureaplasma* species with PID [8, 11].

Furthermore, genitourinary tract infections by *Mycoplasma* and *Ureaplasma* species were reported to be associated with failure of reproductive organs, neonatal morbidity and mortality, and adverse pregnancy outcomes. All of these clinical conditions contribute to infertility [12, 13].

This study was carried out to investigate the prevalence of *M. hominis*, *M. genitalium,* and *U. urealyticum* infections among men and women with infertility and treated at Al-Shunar Center in Nablus. The detection of infection by these pathogens was performed using the PCR method.

## 2. Methods

### 2.1. Design

The present research is a cross-sectional study designed to determine the prevalence of *M. hominis*, *U. urealyticum*, and *M. genitalium* genital infections among infertile male and female patients. The study protocol was approved by IRB at An-Najah University and consent was obtained from each patient.

### 2.2. Patients and Specimens' Collection

The study included patients with infertility visiting Al-Shunar Infertility Center in Nablus city during January 2018 to October 2018, and infertility was not found to be due to hormonal or genetic syndrome causes. Male patients were not included in the study if they suffered from varicocele, testicular torsion, hydrocele, undescended testes, hormonal disorders, or genetic syndrome. Females were not included if they have hormonal disorders or genetic syndrome.

Vaginal swabs, semen, and urine specimens were collected from the included cases. Regardless of the specimen type, at least one specimen was obtained from each patient. Specimen collection process was carried out from January 2018 to October 2018. The semen and first-voided urine specimens were collected into sterile containers and stored at 4°C for short time until they were transported to the laboratory and placed in a sterile Eppendorf tube and stored at −20°C until time of DNA extraction (repeated freezing was avoided).

A sterile cotton swab was used to collect a sample from vagina. The sample was then placed in 3 mL sterile normal saline and stored at 4°C for short period until it was transported to the university laboratory, where it was placed in a sterile Eppendorf tube and stored at −20°C until use.

### 2.3. DNA Extraction

DNA extraction of urine and vaginal swabs was made according to [14]. A volume of 1 mL of the sample was subjected to centrifugation at 12000 × *g* for 10 min. The supernatant was discharged and the pellet was washed with phosphate buffered saline (pH 7.4) and resuspended in 50 *µ*L of distilled water, which was placed in a boiling water bath for 10 min.

DNA extraction from semen specimens was made using QIAamp DNA Mini Kit (QIAGEN, Germany).

The DNA concentration was measured using a spectrophotometer (Jenway 7315, England) using wavelength of 260 nm and a quartz cuvette.

### 2.4. Polymerase Chain Reaction to Detect Studied Mollicutes

The applied oligonucleotide primers sequences were corresponding to sequences of 16S rRNA gene (16S rDNA gene) within the *M. genitalium, U. urealyticum,* and *M. hominis*. The *M. genitalium* primers were 5′-CCT TAT CGT TAG TTA CAT TGT TTA A-3′ and 5′-TGA CAT GCG CTT CCA ATA AA-3′; *U. urealyticum* primers were 5′-ACTATATTTCTA TAG CGTCGCAA-3 and 5′-TACCCTTAAGTT GG GGATAA-3′; and *M. hominis* primers were 5′-ACCCATTGGAAACAATGGCTAATGCCGG-ATACG-3′ and 5′-ATAGACCCAGT AAGCTGCCTTCGCCT-3′ [8, 15].

Each PCR reaction (50 *µ*L) for one sample consisted of 0.1 *µ*M forward primer, 0.1 *µ*M reverse primer, 100 ng/50 *µ*L DNA template, 0.4 mM dNTP, 2 mM MgCl_2_, and 2.5-unit Taq DNA polymerase [8, 15]. The components of PCR reaction were obtained from Sigma (USA) and Invitrogen (USA). The cycling started with an initial denaturation step of five minutes at 94°C, followed by 41 cycles of denaturation at 94°C for one minute, suitable annealing temperature for 30 seconds, and extension for 2 minutes at 72°C. This was followed by a final extension step of five minutes at 72°C. The annealing temperatures for *U. urealyticum*, *M. genitalium*, and *M. hominis* were 46, 46, and 48°C, respectively. Products were electrophoresed in 1.5% agarose (Invitrogen, USA) alongside a 100 bp ladder (Sigma, USA) and stained with ethidium bromide. A UV transilluminator was used to observe DNA bands.

Following detection of each *Mollicutes* by PCR, the samples with positive results for *Mollicutes* were used as a positive control and made together with examined samples in each PCR run. All samples with negative results were retested again with samples with positive results as positive control. In addition, each run of PCR included a negative control.

Nucleotide sequences were determined for representative PCR products to confirm the identity of the studied *Mollicutes*. DNA sequencing was carried out at biological laboratory in Bethlehem University in Palestine. DNA sequencing was made for two PCR products of *U. urealyticum*, one *M. genitalium*, and one *M. hominis*.

### 2.5. Statistical Analysis

Statistical analysis was conducted by a specialist using SPSS for windows. Analysis was carried out to determine relationship between variables (such as gender, age, miscarriage, period of infertility, and smoking tobacco) and the presence of *Mollicutes* infection among the included patients. In addition, the presence of co-infection was also statistically evaluated. A *P* value <0.05 was considered statistically significant.

## 3. Results and Discussion

### 3.1. Polymerase Chain Reaction Results

The present study represents the first investigation of prevalence of *U. urealyticum*, *M. genitalium*, and *M. hominis* in patients diagnosed with infertility in the west bank in Palestine. A total of 110 specimens (16 vaginal swabs, 57 semen, and 37 urine) were collected from 103 patients treated at Al-Shunar Center. Among 110 examined specimen, 35 were PCR positive for at least one *Mollicutes* (including *U. urealyticum, M. genitalium*, and *M. hominis*). In more details, *U. urealyticum* was detected in 11 specimens (10%) out of the 110 specimens, where the expected band (898 bp) was detected. The DNA of *M. genitalium* (343 bp band) was detected in 8 (7.3%) specimens and *M. hominis* DNA (603 bp band) was detected in 16 (14.6%).

In other studies, *M. hominis*, *U. urealyticum*, and *M. genitalium* were detected in 5–40%, 11–35%, and 3–13.4%, respectively, in patients with infertility problems [5, 9–12, 16].

The current study included 6 conjugal partners. *Mollicutes* were not detected in any specimen collected form 4 conjugal partners. In one conjugal partner, *M. hominis* was detected only in the urine specimen of males. In another conjugal partner, *U. urealyticum* was detected in urine specimen of the male and vaginal swab of the female, indicating possible transmission of *U. urealyticum* between conjugal partners.

Table 1 displays PCR results without distinguishing between specimen types collected from infertile men and women. The frequency of detection of *M. hominis* (14.6%) was the highest. Remarkably, *M. hominis* was significantly associated with *U. urealyticum* (3.6%) (*P*=0.044) and *M. genitalium* (3.6%; *P*=0.005). Gdoura et al. [17] also reported that mixed species of *Mycoplasmas* and *Ureaplasmas* were detected in 6.7% of semen sample. Surface structures of the studied bacterial species might complement each other and hence facilitate adhesion of each other to host cells. In more detail, the first type of *Mollicute* species may have surface molecules that are shaped appropriately to bind to surface molecules of the second species. Thus, when the first type of *Mollicute* species binds to the host tissue, it creates a suitable base for binding of the second species type.

Remarkably, the specimen in which the 3 types of bacteria were detected was collected from a male diagnosed to have symptoms of bacterial infection and his wife had symptoms of infection with repeated (3 times) miscarriage. However, they were not diagnosed because of lack of detection of these bacteria in our region, as they cannot be detected by traditional culture.

Seven patients (4 females and 3 males) gave two different types of samples. One male was negative for all examined types of bacteria. In 2 males, *M. genitalium* was detected in semen and urine in one of them, while *M. hominis* was detected in both specimen of one patient and only in semen of the other. Out of the 4 females, only one had *M. hominis* in both vaginal swab and urine samples.

Table 2 compares the frequency of detection of the studied pathogens in different sample types. PCR detected *Mollicutes* in semen specimens in 23 (40.4%) out of 57 specimens, while in urine specimens, 7 out of 37 specimens (18.9%) were with positive PCR results for *Mollicutes*. In addition, PCR detected *Mollicutes* in 5 out of 16 vaginal swabs (31.3%). Remarkably, the percentage of detection of *Mollicutes* in semen (40.4%) was significantly (*P*=0.023) higher than that in urine (18.9%) and insignificantly higher than that in vaginal swab (31.3%). Higher frequencies of detection of *Mollicutes* in specimens collected from the genital tract (semen and vaginal swab) than in urine specimen collected from urinary tract may reflect the tissue tropism of *Mollicutes* toward the genital system. *M. hominis* was found to be the most prevalent in semen (21.1%) and urine (8.1%) specimens. In vaginal swab specimens, *M. hominis* (6.3%) was found to be less prevalent than *U. urealyticum* and *M. genitalium*, which had the same percentage (12.5%) as shown in Table 2.

Association of both *M. hominis* and *M. genitalium* infection (co-infection) in the semen sample was found to be significant (*P* ≤ 0.001), and the co-infection of *M. hominis* and *U. urealyticum* in urine sample was also significant (*P*=0.013).

The prevalence of *Mollicutes* in urine samples from males (29.4%) was higher than that from females (10%). Higher detection of *Mollicutes* in male urine samples may be due to anatomical difference between the two genders, where in males the urethra is longer and an exit for urine and during ejaculation for semen.

In a previous study [17], among 120 semen samples examined by PCR, the frequency of genital *Ureaplasmas* and *Mycoplasmas* detected in semen samples of infertile men were 19.2% and 15.8%, respectively. Campos et al. [18] reported that the molecular finding among 302 women of vaginal swab of *M. hominis* and *M. genitalium* were 31.8%, 28.1%, respectively, and co-infection of both *Mollicutes* was 4.97%. The report of Takahashi et al. [19] in Japan, which included PCR examination of first-voided urine specimens collected from 100 male, recorded detection of *M. genitalium*, *M. hominis*, and *U. urealyticum* in 1%, 4% and 12%, respectively.

The data of 103 patients suffering from infertility are shown in Table 3. Among these patients, the overall prevalence of primary infertility was 65%, which was much higher than secondary infertility (35%). In parallel to our study, Al-Turki [7] during 2015 found among male and female patients suffering from infertility, 78.9% were diagnosed to have primary infertility.

In our study, the mean age of patients with primary infertility (31.5 ± 8.1 years) was insignificantly lower than that of patients with secondary (33.7 ± 8.4%) infertility. This can be explained by the fact that patients with primary infertility seek treatment after delay of pregnancy following marriage. On the other hand, patients with secondary infertility have a history of pregnancy at least once before and only seek treatment after they have the first baby and they are trying for the second one.

**Figure 1:**
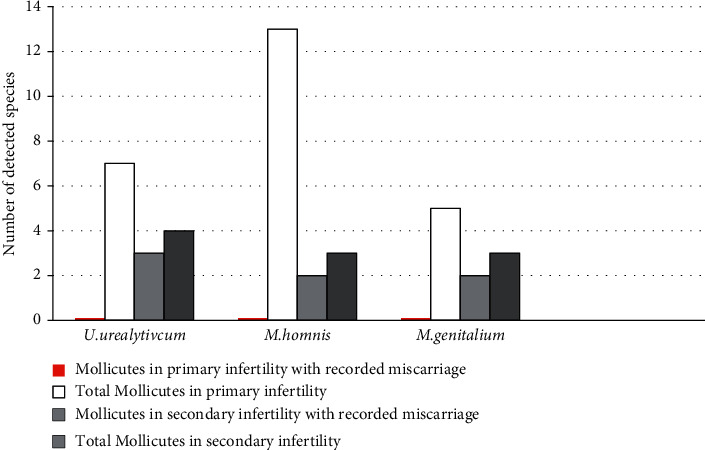
*Mollicutes* distribution among primary and secondary infertile patients.

No correlation was found between age group and type of infertility (*P*=0.503). Our results showed a significant association between period (duration) of infertility and type of infertility, the patients who suffered from secondary infertility had a significantly (*P*=0.028) longer period (7.7 ± 5.7 years) than who suffered from primary infertility (5.5 ± 4.3). In addition, strong significant (*P*=0.000) association between secondary infertility and abortions (miscarriage) was found in the present study. In more detail, among 23 cases of abortions, secondary infertility was recorded in 20 cases. The abortion conditions were significantly associated with patients who had a previous pregnancy, and then they had suffered from infertility problem and were unable to have another child again and the possible reason was having infections after the first pregnancy. Possible etiologies of infections are *Mollicutes* included in our study, where *U. urealyticum*, *M. hominis,* and *M. genitalium* were detected in females with secondary infertility with abortions (miscarriage) in 3, 2, and 2 cases, respectively (Figure 1). Roles of *Mycoplasma* and *Ureaplasma* in causing infertility may be by causing premature rupture of the membranes and inducing of abortion by an inflammatory response by microbial colonization as suggested by other researches [20, 21]. It was also recorded that genital colonization with *M. hominis* and *U. urealyticum* may predispose to spontaneous abortion [22]. A previous study by Maleki et al. [23] revealed a direct strong relationship between *M. hominis* and *U. urealyticum* and habitual abortion as well as urogenital infections. Furthermore, the role of *Mollicutes* in causing miscarriage is supported by treatment results of 5 female patients who had suffered from miscarriage and were included in our research. These patients were *Mollicutes* positive (detected by PCR in our research) and were treated during the process of in vitro fertilization (IVF) by doxycycline monohydrate. The treatment started the first day of IVF protocol for 5 days each day one by two 100 mg of the antibiotic. Out of the 5 female patients, 4 were positive in IVF and continued the pregnancy without any complications.

In the present study, in patients with primary infertility (Table 3), analysis revealed that the highest prevalence of *Mollicutes* infection was that of *M. hominis* (12.6%) followed by *U. urealyticum* (6.8%) and *M. genitalium* (4.9%), whereas *U. urealyticum* (3.9%) possessed the highest prevalence of *Mollicutes* among patients with secondary infertility followed by *M. genitalium* and *M. hominis* (2.9%). This finding was in agreement with the previous report on recurrent (habitual) abortion in the presence of theses *Mollicutes* [23]. Bayoumi et al. [24] also reported that *M. hominis* was detected in 30.4% of women with repeated pregnancy loss and not in pregnant women (as control) and the presence of *M. hominis* was observed more frequently in women with repeated abortions.

In our study, *M. genitalium* infection was significantly associated with females (and not males or both, males and females) diagnosed with secondary infertility, where among 25 female patients with secondary infertility all the 3 PCR positive results were *M. genitalium* (*P*=0.018). Furthermore, *M. genitalium* had significant association (*P*=0.025) among females with a history of miscarriage, where 3 out 11 (27.3%) infertile females suffered from miscarriage were 3 PCR positive for *M. genitalium.* In contrast, no *M. genitalium* was detected in women with no history of miscarriage.

### 3.2. Prevalence of Mollicutes Infections in relation to Different Demographic and Risk Factors

The results presented in Table 4 show the distribution of infection by *Mollicutes* among different gender and age groups. Among the 68 males, 28 (41.2%) possessed PCR positive results for *Mollicutes*. *Mycoplasma hominis* had the highest prevalence (20.6%), followed by *U. urealyticum* (13.2%) and *M. genitalium* (7.5%). The total number of females infected with *Mollicutes* was 7 (20%). Furthermore, males have the highest number of infection by each of the studied pathogen with the exception of *M. genitalium* regardless of specimen types, where only *M. genitalium* infection was more prevalent in females. This high prevalence may be a consequence of histological features of female's vaginal epithelial cells. In vitro studies have demonstrated the ability of *M. genitalium* to rapidly attach to the epithelial cells that line the lumen of the vagina [25].

*Mollicutes* distribution among the 2 genders in our study was different from that in another research, where, in our study, *Mollicutes* were detected in males more than in females. Maleki *et al.* [23] revealed that the highest prevalence of *M. hominis* (71.4%) and *U. urealyticum* (60%) were in females. These differences may be attributed to cultural and religious variations in different regions. In our region, cultural and religious roles have a strong impact on the restriction of sexual relationships of women, where sexual relationships are permitted only through marriage. Men (mostly before marriage) may be engaged in sexual relationships in the nearby countries (where it is permitted) as they can easily travel. As a result, males had higher frequency of urogenital tract infections.

Patients aged between 40–50 years old and 29–39 years old had the highest frequencies of *Mollicutes* positive results, which were 58.3% and 31.9%, respectively. *Mollicutes* were also detected in considerable rates among 18–28 age groups as shown in Table 4. No significant correlation was found between the studied *Mollicutes* and patients with different age groups with the exception of females aged between 18 and 28 years, where a significant association (*P*=0.003) of *M. genitalium* infection was found with them. Its high prevalence could be due to the increase of the genital mycoplasma colonization following puberty. In addition, the prevalence of genital mycoplasma increases after the first sexual contact [26]. This is consistent with our community where the sexual contact as a result of marriage is predominant at age range of 18–28 years old.

The numbers and percentages of occupations of the patients and prevalence of *Mollicutes* among them are also shown in Table 4. Workers have the highest PCR positive results for *Mollicutes* (46.2%), followed by employees (26.5%) and housewives (17.9%). Statistical analysis showed that there was no significant association between patient occupations and frequency of infection by *Mollicutes.* The negative correlation of *Mollicutes* infection may refer to the vulnerability to have this microorganism is increased with sexual activity and risk behaviors and not to economic life and work stress.

Furthermore, *Mollicutes* distributions among smokers (tobacco) varied, where a significant association between tobacco smokers and infection of *M. hominis* (*P*=0.048) was found. In another study, Verteramo et al. [27] reported a significant association between *U. urealtyicum* and smoking (*P*=0.045) in contrast to *M. hominis* infection, which possessed no significant association with smoking.

## 4. Conclusion

Interestingly, *M. hominis*, *U. urealyticum*, and *M. genitalium* appeared to play a possible role in causing infertility among males and females. Although *Mollicutes* infection was detected in all age groups, the *Mollicutes* infection rate was the highest in the age range 40–50 years. *Mollicutes* were detected in both genders where males had higher *Mollicutes* infections than females, and workers had the highest infection rate followed by employees and housewives. Smoking appears to influence the *M. hominis* infection rate.

## Figures and Tables

**Table 1:** PCR results from 110 specimens (semen, urine, and vaginal swabs).

Pathogen	Number	Percentage
	*Single PCR positive result*	

*M. genitalium*	8	7.3
*M. hominis*	16	14.6
*U. urealyticum*	11	10
Total	35	31.8

	*Multiple PCR positive result*	

*M. hominis* *+* *M. genitalium*	4	3.6%
*M. hominis* *+* *U. urealyticum*	4	3.6%
*U. urealyticum* *+* *M. genitalium*	2	1.8%
*M. hominis* *+* *M. genitalium* *+* *U. urealyticum*	1	0.9%

**Table 2:** Detection of *U. urealyticum*, *M. genitalium,* and *M. hominis* in different types of specimens.

Specimen	Number (% out of specimens)	*U. urealyticum* (%)	*M. genitalium* (%)	*M. hominis* (%)	Total number of pathogens (%)
Semen	57 (51.8)	7 (12.3)	4 (7)	12 (21.1)	23 (40.4)
Urine	37 (33.6)	2 (5.4)	2 (5.4)	3 (8.1)	7 (18.9)
Urine male	17 (15.5)	2 (11.8)	1 (5.9)	2 (11.8)	5 (29.4)
Urine female	20 (18.2)	0 (0)	1 (5)	1 (5)	2 (10)
Vaginal swab	16 (14.6)	2 (12.5)	2 (12.5)	1 (6.3)	5 (31.3)
Total	110 (100)	11 (10)	8 (7.3)	16 (14.6)	35 (31.8)

**Table 3:** Comparison between primary and secondary infertility in relation to *Mollicutes* infections.

Parameter	Primary infertility	Secondary infertility
Number of patients	67 (65%)	36 (35%)
Mean age (years)	31.5 ± 0.9	33.7 ± 1.3
Mean period of infertility (years)^*∗*^	5.5 ± 0.5	7.7 ± 0.9
Cases recorded of miscarriage	3 (21.9%)	20 (64.5%)
*M. genitalium*	5	3
	4.9%	2.9%
*M. hominis*	13	3
	12.6%	2.9%
*U. urealyticum*	7	4
	6.8%	3.9%

^*∗*^It is the time (in years) between date of identification of infertility problem and date of collection of specimen from the patient.

**Table 4:** Frequencies of *Mollicutes* infection in relation to different factors.

Variable	Number (%)	*M. hominis* (%)	*U. urealyticum* (%)	*M. genitalium* (%)	Total number of *Mollicutes* (%)
Gender					
Female	35 (34)	2 (5.7)	2 (5.7)	3 (8.6)	7 (20)
Male	68 (66)	14 (20.6)	9 (13.2)	5 (7.4)	28 (41.2)
Age (years)					
18–28	40 (38.8)	5 (12.5)	3 (7.5)	4 (10)	12 (30)
29–39	47 (45.6)	8 (17)	6 (12.8)	1 (2.1)	15 (31.9)
40–50	12 (11.7)	2 (16.7)	2 (16.7)	3 (25)	7 (58.3)
51≥	4 (3.9)	1 (25)	0 (0)	0 (0)	1 (25)
Occupation					
Employee^*∗*^	34 (33)	5 (14.7)	3 (8.8)	1 (2.9)	9 (26.5)
Workers^*∗*^	39 (37.9)	9 (23)	5 (12.8)	4 (10.3)	18 (46.2)
Housewife	28 (27.2)	2 (7.1)	2 (7.1)	2 (7.1)	5 (17.9)
Out of work	2 (1.9)	0 (0)	0 (0)	0 (0)	0 (0)
Smoking					
Yes	48 (46.6)	11 (22.9)	7 (14.6)	5 (10.4)	4 (7.9)
No	55 (53.4)	1 (1.8)	4 (7.3)	4 (7.3)	28 (16.4)

^*∗*^Employee, who works at offices without physical efforts such as a lawyer and marketing manager; workers, who works outside offices with high physical efforts such as builders and painters.

## Data Availability

The data that support the findings and conclusions of this study are included within this article.

## References

[1] Zegers-Hochschild F., Adamson G. D., de Mouzon J. (2009). The international committee for monitoring assisted reproductive technology (ICMART) and the world health organization (WHO) revised glossary on ART terminology. *Human Reproduction*.

[2] Boivin J., Bunting L., Collins J. A., Nygren K. G. (2009). Reply: international estimates on infertility prevalence and treatment seeking: potential need and demand for medical care. *Human Reproduction*.

[3] Row Pj M. A., Comhaire F. H., Hargreavebn T. B. (2000). *WHO Manual for the Standardized Investigation and Diagnosis of the Infertile Malee*.

[4] Abrao M. S., Muzii L., Marana R. (2013). Anatomical causes of female infertility and their management. *International Journal of Gynecology & Obstetrics*.

[5] Inoue S., Tomasini R., Rufini A. (2014). TAp73 is required for spermatogenesis and the maintenance of male fertility. *Proceedings of the National Academy of Sciences*.

[6] Babakhanzadeh E., Nazari M., Ghasemifar S., Khodadadian A. (2020). Some of the factors involved in male infertility: a prospective review. *International Journal of General Medicine*.

[7] Haifa A.-T. (2015). Prevalence of primary and secondary infertility from tertiary center in eastern Saudi Arabia. *Middle East Fertility Society Journal*.

[8] Holmes P. P. K., Sparling P. (2007). *Sexually Transmitted Disease*.

[9] Golshani M. (2007). Detection of *Chlamydia trachomatis*, mycoplasma hominis and Ureaplasma urealyticum by multiplex PCR in Semen sample of infertile men. *Iranian Journal of Public Health*.

[10] Winn W. G., Allen S., Janda W., Koneman E., Procop G., Schreckenrger P., Mycoplasmas (2006). *Konemans Color Atlas and Text Book of Diagnostic Microbiology*.

[11] Simms I. (2003). Associations between Mycoplasma genitalium, *Chlamydia trachomatis*, and pelvic inflammatory disease. *Sexually Transmitted Infections*.

[12] Stellrecht K. A., Woron A. M., Mishrik N. G., Venezia R. A. (2004). Comparison of multiplex PCR assay with culture for detection of genital mycoplasmas. *Journal of Clinical Microbiology*.

[13] Kumar N., Singh A. (2015). Trends of male factor infertility, an important cause of infertility: a review of literature. *Journal of Human Reproductive Sciences*.

[14] Peerayeh S., Sattri M. (2006). Detection of Ureaplasma urealyticum and Mycoplasma hominis in endocervical specimens from infertile women by polymerase chain reaction. *Middle East Fertility Society Journal*.

[15] Ikonomidis A., Venetis C., Georgantzis D. (2016). Prevalence of *Chlamydia trachomatis*, Ureaplasma spp., Mycoplasma genitalium and Mycoplasma hominis among outpatients in central Greece: absence of tetracycline resistance gene tet(M) over a 4-year period study. *New Microbes and New Infections*.

[16] Martens M. G., Young R. L., Uribe M., Buttram V. C., Faro S. (1993). Presence ofChlamydia, mycoplasma, Ureaplasma,and other bacteria in the upper and lower genital tracts of fertile and infertile populations. *Infectious Diseases in Obstetrics and Gynecology*.

[17] Gdoura R., Kchaou W., Chaari C. (2007). Ureaplasma urealyticum, Ureaplasma parvum, Mycoplasma hominis and Mycoplasma genitalium infections and semen quality of infertile men. *BMC Infectious Diseases*.

[18] Campos G. B., Lobão T. N., Selis N. N. (2015). Prevalence of Mycoplasma genitalium and Mycoplasma hominis in urogenital tract of Brazilian women. *BMC Infectious Diseases*.

[19] Takahashi S., Takeyama K., Miyamoto S. (2006). Detection of Mycoplasma genitalium, Mycoplasma hominis, Ureaplasma urealyticum, and Ureaplasma parvum DNAs in urine from asymptomatic healthy young Japanese men. *Journal of Infection and Chemotherapy*.

[20] Rodríguez R., Hernández R., Fuster F., Torres A., Prieto P., Alberto J. (2001). Genital infection and infertility. *Enfermedades Infecciosas y Microbiología Clínica*.

[21] Rosemond A., Lanotte P., Watt S. (2006). Existe-t-il un bénéfice au dépistage systématique de *Chlamydia trachomatis*, Mycoplasma hominis et Ureaplasma urealyticum dans les prélèvements génito-urinaires réalisés au cours d’un bilan d’infertilité?. *Pathologie Biologie*.

[22] Bayraktar M. R., Ozerol I. H., Gucluer N., Celik O. (2010). Prevalence and antibiotic susceptibility of Mycoplasma hominis and Ureaplasma urealyticum in pregnant women. *International Journal of Infectious Diseases*.

[23] Maleki S., Motamedi H., Moosavian S. M., Shahbaziyan N. (2013). Frequency of mycoplasma hominis and ureaplasma urealyticum in females with urogenital infections and habitual abortion history in Ahvaz, Iran; using multiplex PCR. *Jundishapur Journal of Microbiology*.

[24] Bayoumi A., Hussein I., Hind M. (2006). The role of mycoplasmal infection and anticadiolipin antibodies as autoimmune parameters in pregnancy loss. *The Journal of Medical Sciences*.

[25] Mcgowin C. L., Popov V. L., Pyles R. B. (2009). Intracellular Mycoplasma genitalium infection of human vaginal and cervical epithelial cells elicits distinct patterns of inflammatory cytokine secretion and provides a possible survival niche against macrophage-mediated killing. *BMC Microbiology*.

[26] Gupta A., Gupta S., Mittal A., Chandra P., Gill A. K. (2009). Correlation of mycoplasma with unexplained infertility. *Archives of Gynecology and Obstetrics*.

[27] Verteramo R., Patella A., Calzolari E. (2013). An epidemiological survey of Mycoplasma hominis and Ureaplasma urealyticum in gynaecological outpatients, Rome, Italy. *Epidemiology and Infection*.

